# Ancestry variation and footprints of natural selection along the genome in Latin American populations

**DOI:** 10.1038/srep21766

**Published:** 2016-02-18

**Authors:** Lian Deng, Andrés Ruiz-Linares, Shuhua Xu, Sijia Wang

**Affiliations:** 1 Chinese Academy of Sciences (CAS) Key Laboratory of Computational Biology, CAS-MPG Partner Institute for Computational Biology, Shanghai Institutes for Biological Sciences, Chinese Academy of Sciences, Shanghai 200031, China; 2 Department of Genetics, Evolution and Environment, and UCL Genetics Institute, University College London, London, WC1E 6BT, United Kingdom; 3 School of Life Science and Technology, ShanghaiTech University, Shanghai 200031, China; 4 Collaborative Innovation Center of Genetics and Development, Shanghai 200438, China

## Abstract

Latin American populations stem from the admixture of Europeans, Africans and Native Americans, which started over 400 years ago and had lasted for several centuries. Extreme deviation over the genome-wide average in ancestry estimations at certain genomic locations could reflect recent natural selection. We evaluated the distribution of ancestry estimations using 678 genome-wide microsatellite markers in 249 individuals from 13 admixed populations across Latin America. We found significant deviations in ancestry estimations including three locations with more than 3.5 times standard deviations from the genome-wide average: an excess of European ancestry at 1p36 and 14q32, and an excess of African ancestry at 6p22. Using simulations, we could show that at least the deviation at 6p22 was unlikely to result from genetic drift alone. By applying different linguistic groups as well as the most likely ancestral Native American populations as the ancestry, we showed that the choice of Native American ancestry could affect the local ancestry estimation. However, the signal at 6p22 consistently appeared in most of the analyses using various ancestral groups. This study provided important insights for recent natural selection in the context of the unique history of the New World and implications for disease mapping.

The demography in the New World has been dramatically changed since the colonial era. The admixed Latin American populations have received genetic contributions from Native Americans, European immigrants, and Africans largely brought to the New World by the slave trade in the past centuries. Uniquely for the Latin Americans, their admixed genomes inherited from the three continental source populations had to withstand novel environmental challenges in the post-Columbian New World. In principle, when the environment favors genes from one source population, the effects of natural selection will possibly leave detectable signatures in the genomes of the contemporary Latin Americans – an unusual excess of a certain ancestry in the genomic locations of interest, compared to the genome-wide average. While some evidence was reported in studies conducted in a Puerto Rican population^1^, Mexican populations^2^,^3^, Brazilian populations^4^, Tibetans^5^ and African Americans^6^,^7^, there were also criticisms, mainly focusing on three aspects: 1) long range linkage disequilibrium (LD) remaining un-modeled in ancestral populations^8^; 2) inaccurate ancestral populations – Native Americans in particular^9^,^10^,^11^; and 3) the lack of replications^12^.

In this study, we evaluated the distribution of ancestry estimations along the genome using 678 microsatellite markers in samples from 13 admixed populations across Latin America. The microsatellite markers have an average interval of 5 cM, and therefore are less susceptible to the long range LD problem. Unlike previous studies sampling single admixed population, our study covered a wide range of admixed populations in Central and South America, aiming to capture selective forces with universal effect in the continent (e.g. resistance to infectious diseases). We also tested if genetic drift and the choice of Native American ancestry populations could affect the results.

## Results

Since the particular ancestral populations contributing to the Latin American genomes are not known with certainty, we integrated all the African and European samples as the approximate ‘mean’ ancestral pool of Latin Americans. The data we used in this study could not fully represent the populations involved in the admixture about 400 years ago, considering possible genetic drift, natural selection and other demographic events occurred to these populations. STRUCTURE was applied to the genome-wide microsatellite data to estimate the ancestry fractions in the Latin Americans (See Materials and Methods).

**Figure 1:**
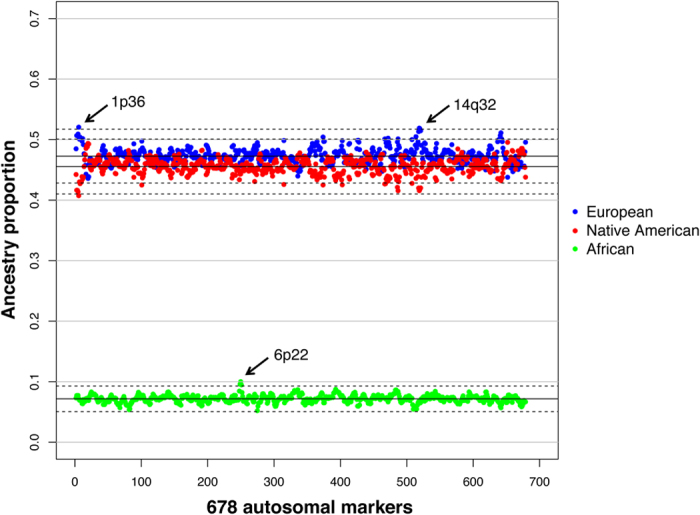
Mean local ancestry admixture estimations for 678 autosomal microsatellite markers of 249 Latin American individuals from 13 populations, by using all 444 Native Americans as ancestry. The solid lines in black indicate the mean ancestry proportion of European, Native American and African, and the dashed lines represent the proportions showing 3.5 standard deviations from the means. The arrows point to three signals: an excess of European ancestry at 1p36 and 14q32, and an excess of African ancestry at 6p22.

By pooling all the Native Americans together as the third ancestral population for the 13 Latin American populations, we estimated the European, African and Native American contributions to the Latin Americans to be 47.27% ± 0.35% (mean ± standard deviation), 6.99% ± 0.14% and 45.56% ± 0.39%, respectively, at population level. Then we averaged the ancestry estimations of the 249 admixed individuals on each locus over the genome. Loci showing strong deviations of a certain ancestry could be the possible candidates of natural selection. The top three signals showing more than 3.5 times standard deviations from the genome-wide average include AAT238 at 1p36 (*Z* score = 3.76, FDR adjusted *P* value = 0.047) and GATA51F04 at 14q32 (*Z* score = 3.63, FDR adjusted *P* value = 0.047) for an excess of European ancestry, and ATA12D05 at 6p22 (*Z* score = 4.67, FDR adjusted *P* value = 0.001) for an excess of African ancestry (Fig. 1). These signals are generally consistent across the 10 replications of admixture analysis with very small variations (see Materials and Methods, and Supplementary Table S1).

It has been shown that a pre- and post-Columbian genetic continuity exists in Latin Americans. The most likely ancestral Native American populations to each of the 13 admixed populations were inferred in Wang *et al*. (2008)^13^. We used the most likely ancestral Native American populations to re-estimate the ancestry proportion for each of the 13 Latin American populations before making an average for each locus over the genome (see Materials and Methods). The average ancestry proportions are 48.14% ± 0.36%, 7.76% ± 0.14% and 44.09% ± 0.36% for European, African and Native American, respectively. These proportions are similar with those estimated using all the Native American ancestral populations. However, the results changed for the three signals of interest, with only ATA12D05 at 6p22 showing significant excessive African influence (*Z* score = 4.71, FDR adjusted *P* value = 8.3 × 10^−4^) (Supplementary Figure S1). Compared with the *Z* scores obtained from simulations under selective neutrality (see Materials and Methods), the *Z* score of ATA12D05 at 6p22 is significant (*P* value = 0.002; Table 1).

**Figure 2:**
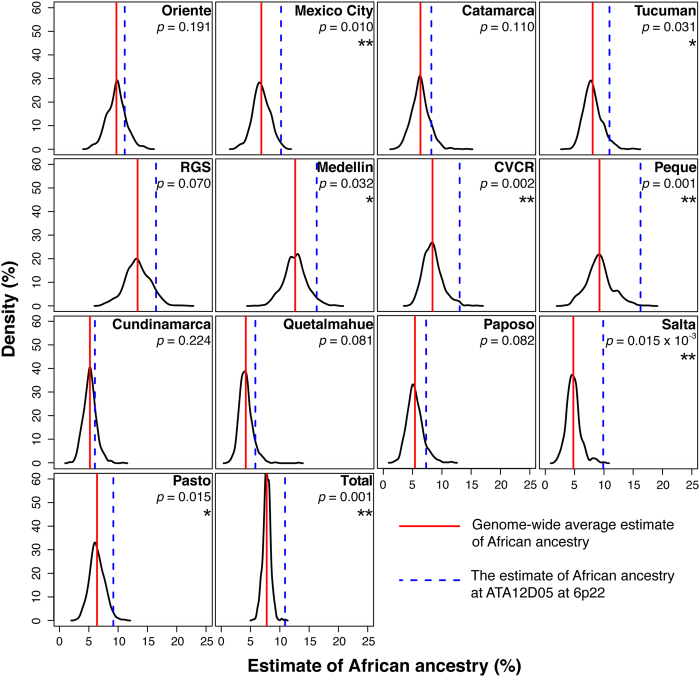
Estimate of African ancestry in 13 Latin American populations. The curve shows the probability density of the African ancestral proportion across the genome. The estimate of African ancestry at ATA12D05 at 6p22 is larger than the genome-wide average in all the 13 populations, among which 7 populations show significant deviations at this locus. Significance at 5% and 1% are indicated by * and **, respectively.

We then used a subset of the Native American dataset – one linguistic stock each time – to represent the Native American ancestral population. The results (Supplementary Figure S2) showed considerably different patterns from what we obtained by using all the Native Americans (Fig. 1) or the most likely Native Americans (Supplementary Figure S1). The proportions of European and African ancestries generally increased at the expense of the Native American proportion. The average ancestry proportions varied across the five analyses using different linguistic stocks (47.92~53.98% for the European ancestry, 7.77~9% for the African ancestry and 37.24~44.31% for the Native American ancestry). As for the three signal loci, only the signal of ATA12D05 at 6p22 remained consistent in three of the five analyses using a single linguistic stock representing the Native American ancestral population (Table 2). Furthermore, the excess of African ancestry appeared in all the 13 Latin American populations when examined alone, among which 7 populations showed significant deviations (Fig. 2). Based on the inference by STRUCTURE, we found allele 131 could have received higher fractions of African ancestry than the other alleles at ATA12D05 at 6p22 in 5 of the 7 Latin American populations (populations from CVCR, Medellin, Mexico City, Peque and Salta). Besides, allele 125 in the populations from Tucuman and Pasto, allele 128 and 140 in the population from Pasto, allele 134 in the population from CVCR, and 146 in the population from Peque also showed significant excess of African influence (Supplementary Table S2). Additionally, by using the non-linkage model, and treating the marker absolutely as independent ones, we found this signal of ATA12D05 at 6p22 remained significant (*Z* score = 5.21, FDR adjusted *P* value = 6.4 × 10^−5^).

The 6p22 region is notably acknowledged to have high genetic diversity and probably under balancing selection. However, in most of the Latin American populations, we did not observe multiple alleles at 6p22 showing significant excess of ancestry proportion from one or more ancestries, which would be expected from balancing selection (Supplementary Table S2). Also in our data, the heterozygosity at 6p22 is not significantly higher than the genome-wide average compared to other genome-wide markers (excess of heterozygosity = 0.05, *Z* score = 0.6, *P* value = 0.27). These findings suggest that the signal at 6p22 inferred from admixture analysis is more likely to be an indication of positive selection, rather than balancing selection. Moreover, this 6p22 region has been previously reported to have an excess of African ancestry in an entirely independent population sample from Puerto Rico^1^, and is also a replication of the signal in Tang *et al*. (2007)^1^. Price *et al*. suggested the possibility of being an artifact, as long range LD was observed in European populations in that region^8^. In the microsatellite data used in our study, no considerable increase of LD was observed in this region in any of the ancestry populations (*P* value > 0.05, Chi-square test for pairwise markers at 6p22).

## Discussion

In our analyses using various Native American ancestral groups, the signal at 6p22 showed consistent excess of African ancestry in the Latin Americans. While the possibility of balancing selection in some populations cannot be entirely ruled out, signatures of positive selection is a better explanation for such observations. By pooling 13 Latin American populations together, we increased the power to detect the signatures of positive selection, but also restrained our targets to the genes with universal selective advantage in the general Latin American populations. For a selective advantage to leave detectable trace in contemporary genomes within about 400 years, the selective force must be extraordinarily strong. In the colonization history, infectious diseases – notably eliminated more than 90% of Native American population within the first one and a half centuries following contact with Europe^14^ – could possibly leave such a mark. Diseases such as smallpox, measles, and influenza caused massive epidemics and were responsible for the extinction of many Native populations^15^,^16^. In this dramatic background, any gene involving infectious disease resistance to gain a selective advantage could leave a well detectable signature in the contemporary Latin American genomes.

Interestingly, the three top signals we discovered all contain genes potentially playing important roles in infectious disease susceptibility or resistance. The first signal at 6p22 is right in the HLA region, where functions of susceptibility or resistance to infectious diseases are well established^17^. The signal at 14q32 is in the region of immunoglobulin heavy chain (*IGH*) gene complex^18^. Around the signal at 1p36, there are several candidate genes, among which *MASP-2* plays an important role in immune defenses against infectious agents^19^,^20^,^21^. Further studies with higher marker density can narrow down the candidate genes, and provide great insight into our understanding of relationship between infectious diseases and natural selection happening in the American continent.

Notably, genetic drift may also cause the excess of ancestry. When population size is small, an allele by chance alone can be inherited more frequently from one ancestry than the other. However, the putative signal of positive selection at 6p22 identified in this study could hardly be explained by genetic drift alone. On one hand, our simulation results clearly showed that the significant excess of local ancestry at 6p22 is no longer detectable under selective neutrality. On the other hand, the early admixture of the 13 Latin American populations were seen as isolated events^22^,^23^. It is very unlikely for genetic drift, which could go both directions, to affect multiple independent samples to the same direction.

This study also demonstrated the importance of the choice of Native American ancestry in locus-specific admixture analysis. Using different ancestry populations could lead to inconsistent findings. Among the three regions reported in this study, 1p36 and 14q32 showed inconsistent signals in different analyses; only 6p22 remained significant when using the most likely Native American ancestral populations and multiple linguistic groups. Therefore, the choice of Native American ancestry is a very likely reason why previous studies had discrepant results. It should be pointed out that the Native American populations used in the current study may have been exposed to European gene flow^24^. This might produce a systematic bias in local ancestry estimations – an overestimated proportion of the Native American and an underestimated European proportion^11^,^25^. But such a systemic bias should affect the results evenly throughout the genome, and therefore would not undermine our main conclusion based on the excess of local ancestry.

One limitation of this study (and similar studies) is the reliance on simplified demographic models. To reflect the most likely colonization history, we constructed a two-stage admixture model to simulate the 13 populations separately, and chose their respective most likely Native American ancestries, admixture time and proportions inferred from real data. While the simulation model is by no means sophisticated enough to reconstruct the fine history of admixture in Latin Americans, it should be sufficient to support the main conclusion on the signatures of positive selection over the genome.

In summary, this study provided insight into adaptive evolution of Latin Americans in the context of the unique history of the New World. It also highlighted the importance of using appropriate ancestral Native American populations in local ancestry inference. In the future, more comprehensive data, by increasing the genomic marker density as well as the population sampling density, will improve our understanding of the genetics and evolutionary history in Latin America.

## Materials and Methods

### Population samples and data

We examined 249 individuals from 13 admixed populations across Latin America, together with 160 Europeans from 8 populations, 123 Africans from 7 populations, and 463 Native Americans from 26 populations as ancestries. All the European and African samples and 5 of the 26 Native American populations are from HGDP-CEPH human genome diversity panel database (v 1.0) (http://www.cephb.fr/hgdp-cephdb/)^26^. The other 21 Native American populations have been reported in Wang *et al*. (2007)^27^. And the 249 Latin American samples were collected from previous population genetic studies and disease association studies (as controls)^28^,^29^,^30^,^31^,^32^,^33^. All the samples were collected with their consent. Ethical approval for the study was provided by the UCL/UCLH ethics committee (UK) as well as by ethnic committees in the countries where the samples were collected. In addition, all the procedures were carried out following the Helsinki Declaration of 1975 (revised in 2000). The geographic and demographic information of all the samples have been detailed in Wang *et al*. (2007)^27^ and Wang *et al*. (2008)^13^. We excluded 19 Native American individuals from the dataset, as they were most likely European immigrants or having an immigrant parent (inferred from STRUCTURE supervised run with GENSBACK = 1, and MIGRPRIOR = 0.001). The final dataset therefore consisted of 444 Native American individuals.

The genotyping of markers was performed by the Marshfield Foundation Mammalian Genotyping Service (http://research.mashfieldclinic.org/genetics/). After removing problematic markers and integrating data with different sources as Wang *et al*. (2007)^27^ and Wang *et al*. (2008)^13^, finally we got 678 microsatellite markers for the subsequent analyses.

### Admixture analysis

We obtained multipoint locus-specific ancestry estimations based on the inter-marker distances specified in the Marshfield map, by using the linkage model implemented in STRUCTURE 2.0 34. Except for the model choice, run conditions were the same as reported in Wang *et al*. (2008)^13^, with data from ancestral populations pooled into K = 3 predefined clusters (Europeans, Africans, and Native Americans). Replicate runs of STRUCTURE used a burn-in period of 20,000 iterations followed by an additional 10,000 iterations from which parameter estimations were obtained. Ten replicate runs were carried out and the average parameter estimation retained.

We tested the robustness of using different Native American ancestral populations, by using only a subset of the available Native American samples as the ancestral population. We used one linguistic stock (as reported in Wang *et al*. (2007))^27^ at each time. Furthermore, to optimize for the most likely Native Americans for each admixed population, we picked the linguistic stock(s) counting for the largest proportion of the Native American ancestry (until the accumulative proportion is over 50% of the overall Native American proportion), based on admixture results shown in Figure 5 of Wang *et al*. (2008)^13^. Each Latin American population was analyzed independently using its closest Native American ancestry, and the ancestry proportion for the total Latin American population was the weighted mean across the 13 populations. We carried out Z test to evaluate the statistical significance for each signal, by assuming normal distribution of locus-specific ancestry estimation over the genome. This assumption is supported by the approximate normal distributions shown in Supplementary Figure S3 (Shapiro-Wilks Test, *P* value > 0.05). The *P* values were corrected by FDR adjusted multiple testing, which was performed using R version 2.11.1 (http://www.r-project.org/)^35^. We strictly tested 678 independent hypotheses corresponding to the 678 markers analyzed, in case that an insufficient correction may lead to false positive signals^12^. The test of LD was performed using Arlequin version 3.5 (http://cmpg.unibe.ch/software/arlequin3/)^36^.

### Simulations

To evaluate the significance of the *Z* score outliers in the admixture analysis, we first performed forward-time simulations based on the Hybrid Isolation (HI) admixture model described in Jin *et al*. (2012)^37^, assuming constant population size for the admixed population (Supplementary Figure S4 for the scheme of the simulation). The 160 European, 123 African samples and the most likely ancestral Native American populations of each of the 13 Latin American populations in the empirical dataset were taken as ancestry populations. The individual genomes of Latin American population were constructed by admixing the genomes of the above three ancestry populations with subsequent recombination events. Mutations were introduced in the simulation based on the stepwise mutation model^38^, assuming that a mutation randomly alters the length of a repetitive array through the addition or removal of one repeat unit at a fixed rate (1 × 10^−4^ per locus per gamete per generation). The admixture of ancestry populations followed the proportions estimated in the supervised STRUCTURE analysis for the real data. We standardized the proportions because we took a two-stage admixture strategy, which means that Europeans mixed with Native Americans first, and one generation later, the individuals generated in the first step mixed with Africans. The number of generations since the second admixture was set differently, ranging from 6 to 14, for the 13 Latin American populations according to Supplementary Table S3 in Wang *et al*. (2008)^13^. The genomes of Latin Americans in the final dataset were randomly sampled from all the simulated individuals to match the sample size of the real data. We then performed locus-specific ancestry estimations on this simulated dataset. Supplementary Figure S5 shows that the simulation fit the data well. We carried out 1,000 independent replicate simulations for each of the 13 Latin American populations, and used the highest *Z* score of each run to construct a *Z* score distribution under neutrality, compared with which the *Z* score of the detected signals obtained their respective *P* values.

## Additional Information

**How to cite this article**: Deng, L. *et al*. Ancestry variation and footprints of natural selection along the genome in Latin American populations. *Sci. Rep.* **6**, 21766; doi: 10.1038/srep21766 (2016).

## Supplementary Material

Supplementary Information

## Figures and Tables

**Table 1:** Comparisons of the top 3 signals with the most significant loci identified under selective neutrality.

Marker	Position	Excess ofAncestral	*Z* score in realdata	Top *Z* score insimulation	*P*value
AAT238	1p36	European	3.00	3.37 ± 0.50	0.770
GATA51F04	14q32	European	3.35	3.37 ± 0.50	0.516
ATA12D05	6p22	African	4.71	3.23 ± 0.52	0.002

The averaged *Z* scores and their standard deviations are described in the fifth column. The *P* value of each signal is calculated based on *Z* test, by assuming normal distribution of the highest *Z* score over 1000 simulations using the most likely ancestral Native Americans.

**Table 2:** Excess of ancestry at AAT238 at 1p36, GATA51F04 at 14q32 and ATA12D05 at 6p22 using each of the 5 linguistic groups as Native American ancestry.

Population	AAT238		GATA51F04		ATA12D05	
	AncestryProportion	*Z* score	AncestryProportion	*Z* score	AncestryProportion	*Z* score
Andean	0.5092	2.3489	0.5225	3.3934*	0.1096	4.6784**
Central Amerind	0.5651	1.5179	0.5501	0.6188	0.1208	4.0313*
Chibchean Paezan	0.5558	2.2653	0.5612	2.5946	0.1231	4.5593**
Equatorial-Tucanoan	0.5847	3.2489	0.5296	2.5913	0.1187	3.5116
Northern Amerind	0.5441	2.5966	0.5211	1.5919	0.1065	3.6235

FDR adjusted *P* values significant at the 0.05 level are marked with *, and those significant at the 0.01 level are marked with **.
