# Supplementary material for: Ancestry variation and footprints of natural selection along the genome in Latin American populations
Source: Sci Rep. 2016 Feb 18;6:21766. doi: 10.1038/srep21766 (PMC4757894; doi:10.1038/srep21766)
Supplement: Supplementary Information [file srep21766-s1.doc]

**Ancestry variation and footprints of natural selection along the genome in Latin American populations**

Lian Deng^1^, Andres Ruiz-Linares^2^, Shuhua Xu^1,3,4,*^, Sijia Wang^1,4,*^

^1^Chinese Academy of Sciences (CAS) Key Laboratory of Computational Biology, CAS-MPG Partner Institute for Computational Biology, Shanghai Institutes for Biological Sciences, Chinese Academy of Science, Shanghai 200031, China;

^2^Department of Genetics, Evolution and Environment, and UCL Genetics Institute, University College London, London WC1E 6BT, United Kingdom;

^3^School of Life Science and Technology, ShanghaiTech University, Shanghai 200031, China;

^4^Collaborative Innovation Center of Genetics and Development, Shanghai 200438, China.

*Corresponding to: [xushua@picb.ac.cn](mailto:xushua@picb.ac.cn) or [wangsijia@picb.ac.cn](mailto:wangsijia@picb.ac.cn)

**Supplementary Information**

**Figure Legends**

**Supplementary Figure S1. Mean local ancestry admixture estimations for 678 autosomal microsatellite markers of 249 Latin American individuals from 13 populations, by using the closest regional Native American populations as ancestry.**

The closest regional Native American populations for each Latin American population are defined by following the description in Materials and Methods. The solid black lines indicate the mean ancestry proportion of European, Native American and African, and the dashed lines represent the proportions showing 3.5 standard deviations from the means. The arrows point to the signals at 6p22 showing an excess of African ancestry.

**Supplementary Figure S2. Mean local ancestry admixture estimations for 678 autosomal microsatellite markers of 249 Latin American individuals from 13 populations, by using one Native American linguistic stock as ancestry at a time.**

Following Wang et al. (2007) (ref. 27), the Native Americans are grouped into Central Amerind, Northern Amerind, Equatorial-Tucanoan, Andea, and Chibchean Paezan. The solid lines in black indicate the mean ancestry proportion of European, Native American and African, and the dashed lines represent the proportions showing 3.5 standard deviations from the means.

**Supplementary Figure S3. Distribution of local ancestry estimations of 678 microsatellite markers across the genome.**

The distributions of African and European ancestry estimations are shown in the histograms A and B, respectively. Bin size is 0.616. Only one count each from African and European ancestry estimations are out of the 4 standard deviations range (ATA12D05 at 6p22 in African ancestry estimations and GATA045 at 14q32 in European ancestry estimations). The grey areas in the background are the normal distribution shapes constructed using the 677 *Z* scores in the range of [-4, 4].

**Supplementary Figure S4. Scheme of simulation of 13 Latin American populations.**

The time of admixture are given according to Wang et al., (2008) (ref. 13). The admixture of ancestry populations followed the proportions estimated in the supervised STRUCTURE analysis for the real data. n_1_ and n_2_ represent the sample size of the closest ancestral Native Americans of each admixed population and the sample size of the simulated admixed population, respectively. p_1_, p_2_, p_3_, and p_4_ denote the proportions of admixture. Details of these parameters can be found in the table below.

**Supplementary Figure S5. Local ancestry admixture estimations for 678 autosomal microsatellite markers of 249 Latin American individuals from 13 simulated populations, by using the closest regional Native American populations as ancestry.**

The 249 Latin American individuals were simulated following the procedure showing in Supplementary Figure S5. Here we randomly selected one repeat of simulation from 1000, and the proportion estimated using simulated data is consistent with that using the real samples in supplementary Figure S1. The solid lines in black indicate the mean ancestry proportion of European, Native American and African, and the dashed lines represent the proportions showing 3.5 standard deviations from the means.

Supplementary Figure S1


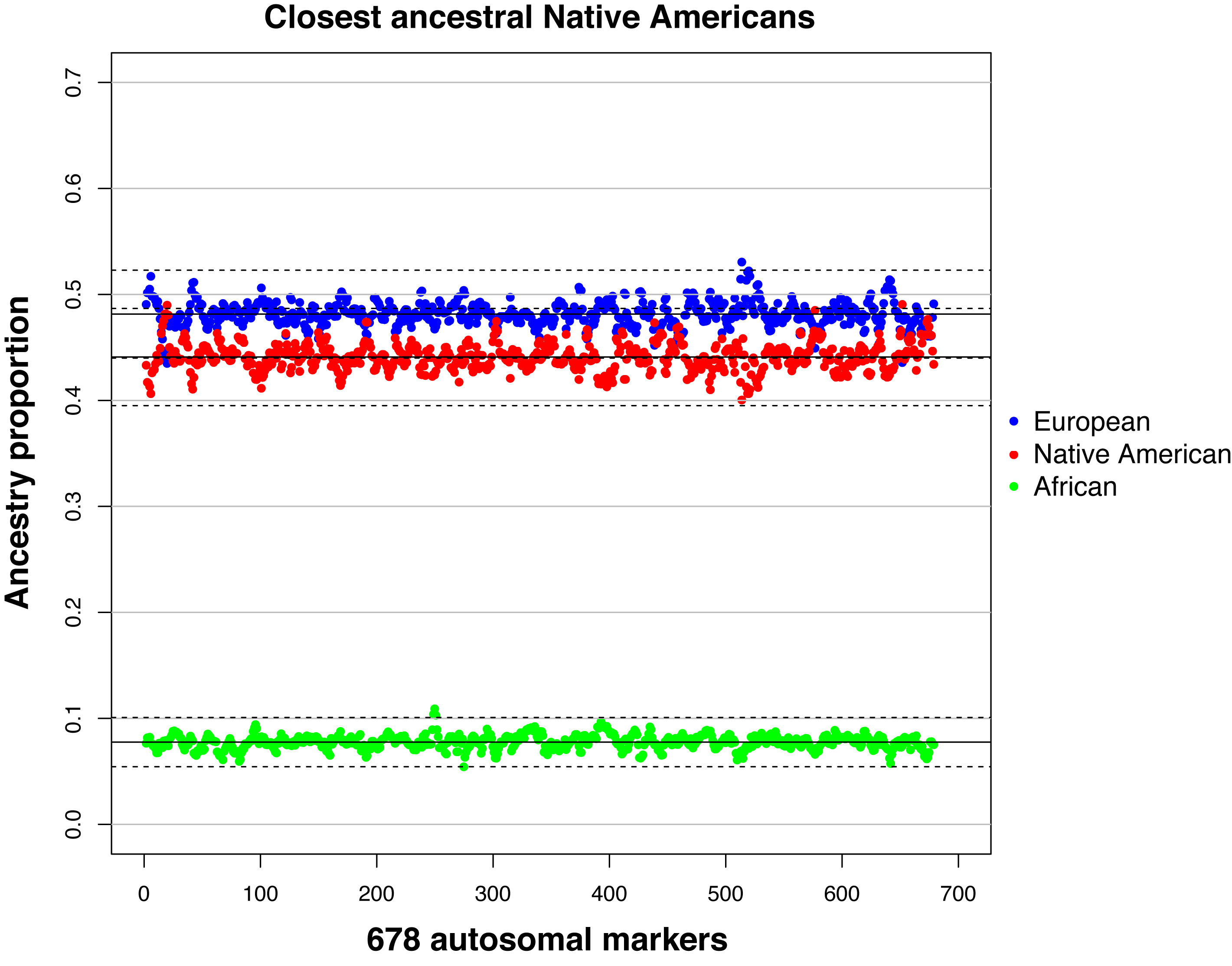


6p22

Supplementary Figure S2


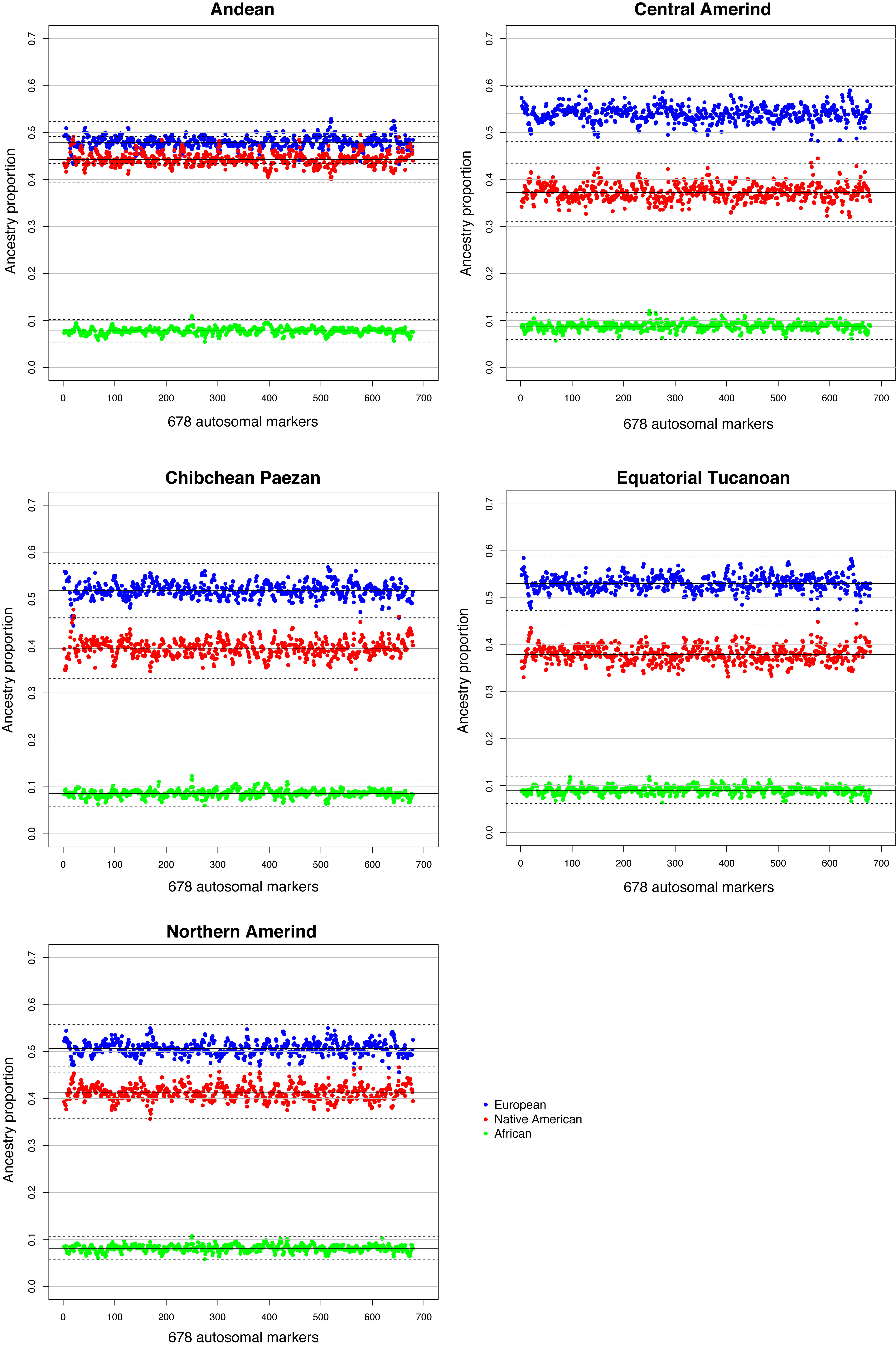


Supplementary Figure S3

Supplementary Figure S4

| **Latin American** | **Ancestral Native American** | **g** | **n1** | **n2** | **p1** | **p2** | **p3** | **p4** |
| --- | --- | --- | --- | --- | --- | --- | --- | --- |
| Oriente | Northern Amerind + Andean | 9 | 124 | 20 | 41.8 | 58.2 | 90.4 | 9.6 |
| Mexico City | Northern Amerind + Andean | 9 | 124 | 19 | 55.9 | 44.1 | 93.2 | 6.8 |
| Catamarca | Northern Amerind + Andean | 11 | 124 | 14 | 52.6 | 47.4 | 93.7 | 6.3 |
| Tucuman | Northern Amerind + Andean | 10 | 124 | 19 | 65.5 | 34.6 | 91.9 | 8.1 |
| RGS | Northern Amerind + Andean + Equatorial-Tucanoan | 7 | 259 | 20 | 74.9 | 25.1 | 86.7 | 13.3 |
| Medellin | Northern Amerind + Chibchean Paezan | 10 | 170 | 20 | 70.5 | 29.6 | 87.4 | 12.6 |
| CVCR | Northern Amerind + Chibchean Paezan | 14 | 170 | 20 | 67.9 | 32.1 | 91.6 | 8.4 |
| Peque | Chibchean Paezan | 8 | 115 | 20 | 43 | 57 | 90.8 | 9.2 |
| Cundinamarca | Chibchean Paezan + Andean | 12 | 184 | 19 | 48.1 | 51.9 | 94.9 | 5.1 |
| Quetalmahue | Andean | 9 | 69 | 20 | 48.1 | 51.9 | 95.9 | 4.1 |
| Paposo | Andean | 8 | 69 | 20 | 43.2 | 56.8 | 94.6 | 5.4 |
| Salta | Andean | 6 | 69 | 19 | 28.3 | 71.7 | 95.2 | 4.8 |
| Pasto | Andean | 9 | 69 | 19 | 41.5 | 58.5 | 93.7 | 6.3 |

Supplementary Figure S5


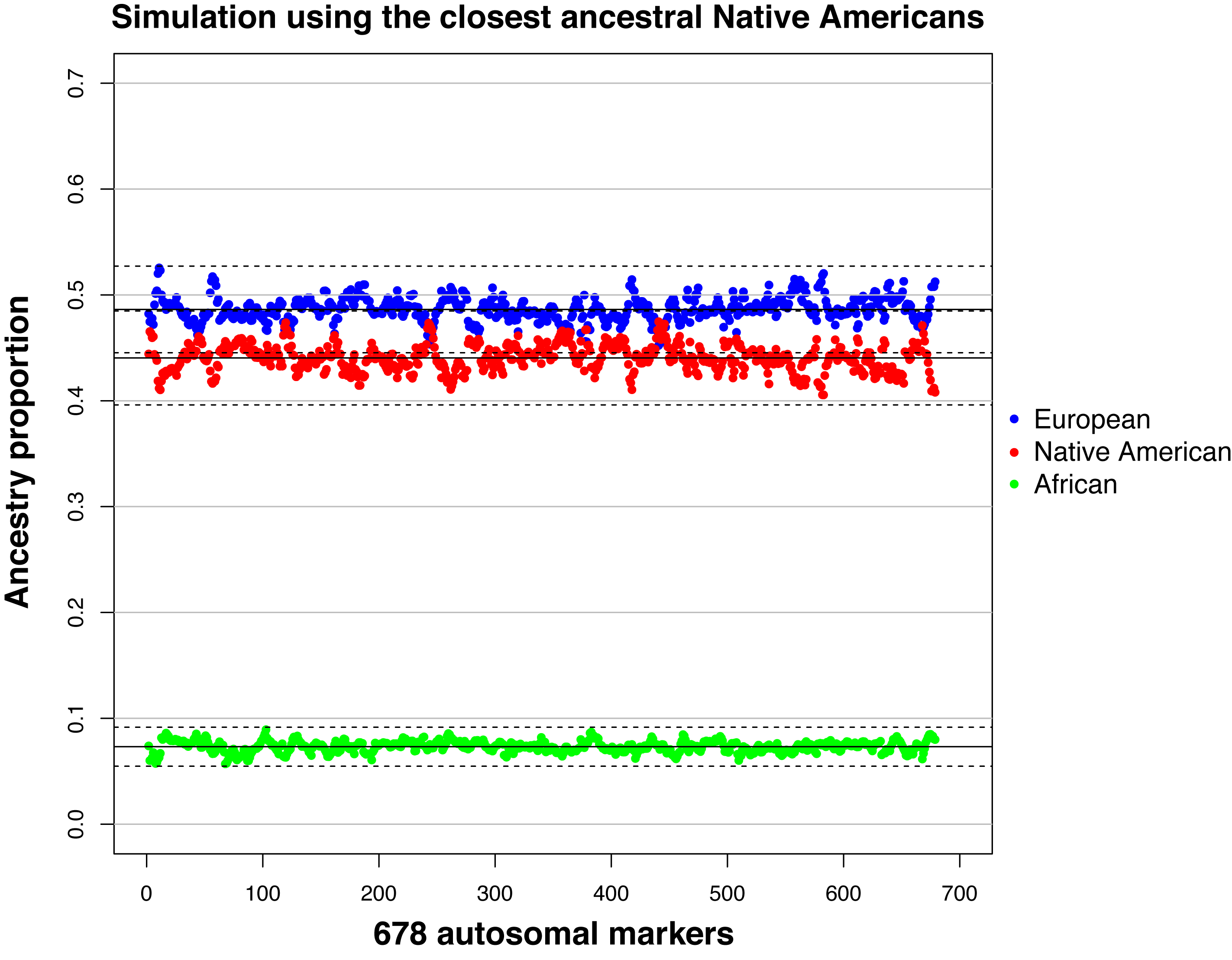


Table S1. The top 3 loci showing most significant signals identified using all 444 Native Americans as ancestry.

| Marker | Position | Excess of Ancestry | *Z* score | *P* value | Ten replications of admixture analysis | | |
| --- | --- | --- | --- | --- | --- | --- | --- |
|  |  |  |  |  | Mean Excess | Standard Deviation of Excess | Consistency of Signals (*P* value < 0.05) |
| AAT238 | 1p36 | European | 3.76 | 0.047 | 0.048 | 0.001 | 8 |
| GATA51F04 | 14q32 | European | 3.63 | 0.047 | 0.046 | 0.01 | 6 |
| ATA12D05 | 6p22 | African | 4.67 | 0.001 | 0.028 | 0.004 | 9 |

The *P* value of each signal is calculated for the mean excess of the 10 replications of admixture analysis (see Materials and Methods) on each marker, and it is based on *Z* test assuming normal distribution of the genome-wide estimations. The last column indicates the number of replications showing signals at each of the three loci.

Table S2. Deviation of African ancestry at ATA12D05 at 6p22

| Allele | Oriente | Mexico City* | Catamarca | Tucuman* | RGS | Medellin* | CVCR* | Peque* | Cundina-marca | Quetalm-ahue | Paposo | Salta* | Pasto* |
| --- | --- | --- | --- | --- | --- | --- | --- | --- | --- | --- | --- | --- | --- |
| 125 | - | - | - | **0.747** | - | - | - | - | - | - | - | - | **0.578** |
| 128 | - | - | - | - | - | - | - | - | - | - | - | - | **0.386** |
| 131 | **0.692** | **0.671** | **0.715** | - | **0.648** | **0.798** | **0.489** | **0.718** | - | **0.720** | **0.760** | **0.672** | - |
| 134 | -0.003 | 0.000 | - | 0.072 | 0.100 | 0.131 | **0.210** | -0.088 | **0.126** | 0.035 | 0.087 | 0.029 | -0.032 |
| 137 | -0.021 | -0.056 | -0.036 | -0.057 | -0.122 | -0.080 | -0.085 | -0.091 | -0.041 | -0.024 | -0.039 | -0.024 | -0.051 |
| 140 | -0.047 | -0.074 | -0.032 | -0.015 | -0.056 | -0.056 | -0.019 | -0.079 | **0.030** | -0.028 | -0.025 | -0.069 | **0.086** |
| 143 | -0.057 | -0.043 | -0.004 | -0.025 | -0.031 | -0.040 | -0.056 | -0.075 | -0.026 | -0.026 | -0.024 | -0.060 | -0.033 |
| 146 | 0.033 | -0.015 | -0.019 | 0.090 | 0.079 | 0.044 | 0.045 | **0.178** | 0.013 | -0.014 | -0.005 | -0.057 | -0.026 |
| 149 | -0.047 | -0.051 | -0.065 | -0.061 | -0.110 | -0.068 | -0.093 | -0.112 | -0.018 | -0.031 | -0.004 | -0.045 | -0.019 |
| 152 | -0.007 | -0.077 | -0.050 | - | - | - | - | - | - | - | - | - | -0.036 |

Latin American populations showing significant excess of African ancestry at 6p22 are indicated by asterisks. Significant deviations (*P* value < 0.05, Permutation test) are highlighted in bold.
